# Functional cooperation between LIPI-4 and listeriolysin O governs intracellular replication and systemic virulence of *Listeria monocytogenes*

**DOI:** 10.3389/fmicb.2026.1853120

**Published:** 2026-07-17

**Authors:** Zhongke Yin, Zhijie Luo, Xun Ma, Caixia Liu, Yatao Qi, Lu Liu, Ruixuan Qian, Jing Wang, Guangdong Hu

**Affiliations:** College of Animal Science and Technology, Shihezi University, Shihezi, China

**Keywords:** *hly*, LIPI-4, *Listeria monocytogenes*, listeriolysin O (LLO), virulence

## Abstract

**Background:**

*Listeria monocytogenes* (*L. monocytogenes*) is a facultative intracellular bacterium and the etiological agent of listeriosis, an invasive infection that disproportionately affects neonates, older adults, pregnant women, and immunocompromised individuals. Among the major virulence determinants of *L. monocytogenes*, listeriolysin O (LLO) and the pathogenicity island LIPI-4 have been implicated in intracellular survival and tissue tropism. Here, we examined whether LLO contributes to LIPI-4-associated pathogenicity. While both LIPI-4 and LLO are known to be important for *L. monocytogenes* virulence, it remains to be determined whether LIPI-4 promotes infection by modulating LLO expression and secretion.

**Methods:**

Using the frozen raw chicken isolate LM928 as the parental strain, we constructed an *hly* deletion mutant (LM928Δ*hly*), an *hly*/LIPI-4 double-deletion mutant (LM928ΔLIPI-4/*hly*), and the corresponding complemented strains via homologous recombination. Genetic stability was evaluated by serial passaging followed by PCR confirmation. LLO expression was assessed by Western blotting, and bacterial growth kinetics and hemolytic activity were examined *in vitro*. Adhesion, invasion, and intracellular replication were assessed in human cerebral microvascular endothelial cells (HCMEC/D3) and placental trophoblast cells (HTR-8). *In vivo* virulence was determined by quantifying bacterial burdens in the brain and spleen of infected mice.

**Results:**

All mutant and complemented strains remained genetically stable, and deletion of *hly* or LIPI-4 had no measurable effect on bacterial growth *in vitro*. LLO was not detected in the *hly* deletion mutant or in the *hly*/LIPI-4 double-deletion mutant. In contrast, the LIPI-4 deletion mutant retained hemolytic activity, although at a reduced level compared with the wild-type strain. In cell infection assays, deletion of LIPI-4 resulted in reduced adhesion, invasion, and intracellular replication in both HCMEC/D3 and HTR-8 cells, and these impairments were greater than those observed for the *hly* mutant. Among all strains tested, the double-deletion mutant showed the most pronounced defects across the *in vitro* assays. In a mouse infection model, deletion of either *hly* or LIPI-4 reduced bacterial burdens in the brain and spleen, and the lowest burdens were observed in animals infected with the double-deletion strain.

**Conclusion:**

Our results indicate that *hly* and LIPI-4 act together to promote *L. monocytogenes* virulence, with LIPI-4 having a stronger impact on host cell interaction and intracellular persistence. These data support a coordinated role for LLO and LIPI-4 during systemic infection and provide insight into mechanisms that may contribute to tissue tropism and barrier crossing by *L. monocytogenes*.

## Introduction

*L. monocytogenes* is a facultative intracellular pathogen that causes listeriosis, a severe foodborne disease that disproportionately affects high-risk groups, including neonates, pregnant women, older adults, and immunocompromised individuals. Notably, *L. monocytogenes* is associated with a high case-fatality rate among foodborne pathogens, reaching approximately 30% (Disson et al., 2025; Lamont et al., 2011). During pregnancy, infection can result in miscarriage, stillbirth, or neonatal death, and a retrospective analysis of 10 pregnancy-associated cases reported between 2015 and 2023 documented a neonatal mortality rate of up to 70% (Fu et al., 2025). Beyond its clinical impact, *L. monocytogenes* is well adapted to environmental stress and can persist under conditions such as low temperature, acidic pH, high salt or bile concentrations, oxidative stress, and nutrient limitation (Nguyen, 2019; Osborne, 2017; Bavdek et al., 2012). Its ability to grow at refrigeration temperatures contributes to contamination of chilled and ready-to-eat foods. In China, a nationwide survey conducted between 2012 and 2014 detected *L. monocytogenes* in approximately 20% of 1,036 retail fresh food samples, indicating that it is widely present in the food supply chain (He et al., 2021; Zheng, 2015). These features highlight the continuing public health relevance of *L. monocytogenes*. Successful infection depends on multiple virulence factors that promote host cell invasion, intracellular survival, and systemic dissemination (Khan, 2025). Among these, the *hly* gene encodes listeriolysin O (LLO), a cholesterol-dependent pore-forming toxin that is required for escape from the phagosome into the host cytosol (Levraud et al., 2009; Jones et al., 2015; Wei et al., 2005; Tilney and Portnoy, 1989). LLO binds cholesterol-rich membrane regions and oligomerizes to form transmembrane pores, thereby damaging endosomal membranes and supporting intracellular survival (Nguyen, 2019; Fields et al., 2025; Christie et al., 2018; Gilbert, 2010).

The virulence of *L. monocytogenes* (LM) is governed by multiple virulence determinants, notably pathogenicity island 4 (LIPI-4) and the *hly* gene, whose encoded proteins contribute to intracellular survival, cell-to-cell spread, and systemic infection. In particular, the *hly* gene encodes LLO, a pore-forming toxin that disrupts host cell membranes, promoting bacterial invasion, intercellular dissemination, and replication-processes central to LM pathogenicity (Levraud et al., 2009; Jones et al., 2015; Wei et al., 2005; Tilney and Portnoy, 1989). Inside host cells, LLO functions as a pivotal cholesterol-dependent cytolysin (CDC) that specifically recognizes and binds to cholesterol-enriched domains of host membranes (Fields et al., 2025). Via its characteristic cholesterol-binding domain, LLO oligomerizes into ring-shaped complexes, establishing the foundation for pore formation across the membrane (Nguyen, 2019). These oligomers then undergo conformational rearrangements, generating large β-barrel transmembrane structures that form pores approximately 30–40 nm in diameter within the membrane (Christie et al., 2018; Gilbert, 2010). The pore formation compromises the integrity of the endosomal membrane, enabling LM to escape into the cytosol of host cells. This mechanism greatly augments the invasiveness and intracellular survival of LM, allowing efficient dissemination within host tissues (Roth et al., 2024).

Several pathogenicity islands have been identified in *L. monocytogenes*, including Listeria pathogenicity island 1 (LIPI-1), Listeria pathogenicity island 2 (LIPI-2), Listeria pathogenicity island 3 (LIPI-3), and Listeria pathogenicity island 4 (LIPI-4) (Mota et al., 2025), which contribute to different aspects of bacterial virulence. Among these pathogenicity islands, LIPI-4 is of particular interest due to its strong association with central nervous system and placental infections, where it plays a key role in mediating tissue-specific tropism and contributing to the pathogenic potential of *L. monocytogenes* (Wiktorczyk-Kapischke et al., 2023a). LIPI-4 is predominantly associated with hypervirulent clonal complex CC4 strains, but has also been reported in other lineages such as CC87 and ST87, indicating that its distribution is not strictly restricted to a single clonal complex (Wiktorczyk-Kapischke et al., 2023b). LIPI-4 consists of six sequential genes (Lm4b_02324, Lm4b_02325, Lm4b_02326, Lm4b_02327, Lm4b_02328 and Lm4b_02329), all functionally linked to the phosphoenolpyruvate (PEP)-dependent phosphotransferase system (PTS) (Liu et al., 2025). Specifically, Lm4b_02324 encodes maltose-6′-phosphate glucosidase, Lm4b_02325 encodes a transcriptional antiterminator, Lm4b_02326 encodes a carbohydrate deacetylase, Lm4b_02327 encodes PTS subunit EIIA, Lm4b_02328 encodes PTS subunit EIIB, and Lm4b_02329 encodes PTS subunit EIIC (Shi et al., 2023). Sequence characterization indicated that this gene cluster mainly participates in carbon metabolism and carbon source sensing via PTS and carbohydrate hydrolases. Only Lm4b_02328 encodes a putative secreted protein, while none of these proteins are predicted to regulate bacterial motility (Nguyen, 2019). The PTS is a highly conserved protein complex widely distributed in bacteria. It mediates the coupled transport and phosphorylation of carbohydrates and acts as a pivotal regulator of bacterial metabolism (Meireles et al., 2024). Beyond metabolic roles, PTS is integrated into global regulatory networks and modulates the expression of virulence-related genes. Accumulating evidence also indicates that carbon metabolism and nutrient sensing are tightly correlated with bacterial virulence. Because LIPI-4 encodes a PTS involved in carbohydrate uptake and metabolic regulation, it may influence virulence-associated pathways through metabolic signaling (Pokorzynski and Groisman, 2023). Previous studies have demonstrated that carbon metabolism and nutrient sensing are closely linked to virulence gene regulation in *L. monocytogenes* (Krawczyk-Balska et al., 2021). Since *hly* is a major target of the PrfA virulence regulon, it is reasonable to hypothesize that LIPI-4-associated metabolic regulation may indirectly influence *hly*/LLO expression. This possibility provides a biological rationale for investigating the relationship between LIPI-4 and LLO in the present study.

Previous studies have associated LIPI-4 with neuroinvasion and placental tropism in *L. monocytogenes*. Maury et al. investigated the LIPI-4-positive strain LM09-00558 in a murine infection model and reported that deletion of the PTS gene cluster (ΔPTS) within LIPI-4 substantially reduced the ability of the bacterium to cross the blood-brain barrier, while having little effect on colonization of other organs (Liu et al., 2025; Shi et al., 2023; Maury et al., 2016). In line with this, LIPI-4 deletion was also shown to decrease invasion and cell-to-cell spread in human brain microvascular endothelial cells, resulting in reduced virulence (Ruan et al., 2023). In addition, recent evidence suggests that loss of LIPI-4 is accompanied by decreased *hly* expression (Maury et al., 2016), raising the possibility of regulatory crosstalk between these virulence loci. Nevertheless, it remains unclear whether LIPI-4 contributes to pathogenicity by modulating LLO expression and activity, and the underlying mechanism has not been fully defined. Taken together, LIPI-4 represents a specialized virulence-associated PTS gene cluster that contributes to neuroinvasion and placental infection, and accumulating evidence suggests that it may influence bacterial pathogenicity through modulation of virulence-associated regulatory networks. Importantly, given that LLO encoded by *hly* represents a central effector of intracellular survival and is tightly regulated by metabolic and environmental cues through the PrfA regulatory network, the potential coupling between a metabolic PTS-encoding island (LIPI-4) and *hly* expression represents a previously unexplored but biologically plausible regulatory axis in *L. monocytogenes*. Therefore, elucidating the relationship between LIPI-4 and LLO may provide new insights into the molecular mechanisms underlying hypervirulence in *L. monocytogenes*.

In this study, we investigated whether LLO contributes to LIPI-4-associated virulence and examined the relationship between LIPI-4 and the *hly* locus. To this end, we generated an *hly* deletion mutant (LM928Δ*hly*), a double mutant lacking both LIPI-4 and *hly* (LM928ΔLIPI-4/*hly*), and the corresponding complemented strains using homologous recombination. These strains were evaluated alongside the parental strain for *in vitro* growth, hemolytic activity, adhesion, invasion, and intracellular proliferation in relevant host cell models. In addition, a mouse infection model was used to assess *in vivo* virulence and tissue colonization. This study therefore provides experimental evidence to define how LIPI-4 and LLO act together during *L. monocytogenes* infection and helps clarify mechanisms that may contribute to tissue tropism and systemic dissemination.

## Materials and methods

### Bacterial strains

The *L. monocytogenes* strain LM928 was isolated and identified by the Food Inspection Center of the Xinjiang Academy of Agricultural Reclamation Sciences and was kindly provided to our laboratory for experimental use. The LM928ΔLIPI-4 deletion strain was previously constructed and characterized in our laboratory as described by Ruan et al. (2023) and was maintained in our laboratory collection.

### Plasmids

The temperature-sensitive shuttle vector pKSV7 was kindly donated by the Laboratory of Molecular Microbiology and Food Safety, College of Animal Sciences, Zhejiang University. The plasmid pIMK2 was kindly provided by Professor Yin Yuelan from the College of Biological Science and Technology, Yangzhou University. The pMD19-T (Simple) cloning vector was obtained from Takara Biomedical Engineering Co., Ltd. (Dalian, China). Anti-listeriolysin O (LLO) rabbit polyclonal antibody (catalog no. ab200538) was purchased from Abcam (Cambridge, UK). CoraLite594-conjugated goat anti-rabbit IgG (H+L) (catalog no. SA00013-4) was purchased from Proteintech (Wuhan, China). Sensitive ECL Chemiluminescence Detection Kit (catalog no. PK10002) was purchased from Proteintech (Wuhan, China).

### Cell lines

The human cerebral microvascular endothelial cell line HCMEC/D3 and the human chorionic trophoblast cell line HTR-8/Svneo were obtained from the BeNa Culture Collection (Xinyang, China).

### Key reagents

DNA ligase and restriction enzymes (BamHI, PstI, and XhoI) were obtained from Takara Biomedical Engineering Co., Ltd. (Dalian, China). *E. coli* DH5α competent cells, 5K DNA marker, RNA extraction kit, and real-time quantitative PCR (qPCR) kit were purchased from Beijing TransGen Biotech Co., Ltd. (Beijing, China). The agarose gel DNA purification kit, plasmid miniprep kit, 2 × Taq Master Mix (DyePlus), DL2000Plus DNA marker, and Pfu DNA polymerase were purchased from Vazyme Biotech Co., Ltd. (Nanjing, China). The bacterial genomic DNA extraction kit was purchased from TIANGEN Biochemical Technology Co., Ltd. (Beijing, China).Ampicillin (Amp), HEPES, chloramphenicol, lysozyme, gentamicin, and kanamycin were obtained from Beijing BioDee Biotechnology Co., Ltd. Brain Heart Infusion (BHI) broth and fermentation tubes were obtained from Hope Bio-Technology Co., Ltd. (Qingdao, China). RNA extraction kit (TransGen Biotech, China).

### Animals and ethics statement

ICR mice aged 6–8 weeks were purchased from SPF Biotechnology (Beijing, China; https://spfbiotech.com, accessed on 20 March 2024). Throughout the experiment, mice were maintained under controlled temperature (21–26 °C) and humidity (40–70%). All animal experimental programs complied with the provisions of the National Guidelines for Foster Care and Care of Experimental Animals (China). All animal experiments were approved by the Bioethics Committee of Shihezi University (Approval Number: A2021-26; Approval date: 10 March 2021).

### Primer design

The *hly* gene sequence was retrieved from the complete genome of the *L. monocytogenes* LM928 strain available in the NCBI database (GenBank Accession No. NZ_CP046478.1). Specific primers were designed using Primer Premier 5.0 software, and the primer sequences for gene amplification and verification of the double-deletion mutants are presented in Table 1. All primers were synthesized commercially by Youkang Biotechnology Co., Ltd. (Xinjiang, China).

**Table 1:** Plasmids used in this study.

Plasmid	Relevant characteristics	Source	Purpose in this study
pKSV7	Temperature-sensitive shuttle vector; chloramphenicol resistance	Kindly provided by the Laboratory of Molecular Microbiology and Food Safety, College of Animal Sciences, Zhejiang University	Allelic exchange vector for construction of deletion mutants
pMD19-T	TA cloning vector	Takara Biomedical Engineering Co., Ltd.	Cloning and sequence verification of PCR fragments
pIMK2	Integrative vector; kanamycin resistance	Kindly provided by Prof. Yuelan Yin, Yangzhou University	Construction of complemented strains
pMD19-T-Δ*hly*	pMD19-T carrying the fused upstream and downstream homologous arms of *hly*	This study	Intermediate plasmid for construction of pKSV7-Δ*hly*
pKSV7-Δ*hly*	pKSV7 carrying the Δ*hly* deletion fragment	This study	Construction of LM928Δ*hly* and LM928ΔLIPI-4/Δ*hly* mutants
pIMK2-*hly*	pIMK2 carrying the *hly* coding sequence	This study	Construction of *hly*-complemented strains

### Construction of the Δ*hly* mutant, ΔLIPI-4/*hly* double-deletion mutant, and complemented strains

#### Construction of the Δ*hly* deletion mutant

Genomic DNA of LM928 was extracted with a bacterial genomic DNA extraction kit and served as the template for PCR. The upstream and downstream homologous arms of the *hly* gene were amplified using specific primers and fused via overlap extension PCR (SOE-PCR) to obtain the Δ*hly* deletion fragment. The fused product was purified and inserted into the pMD19-T vector, followed by sequencing verification. The correctly sequenced fragment was further subcloned into the temperature-sensitive shuttle vector pKSV7 via BamH I and Pst I digestion to construct the recombinant plasmid pKSV7-Δ*hly*, which was subsequently validated by restriction enzyme digestion and sequencing. The recombinant plasmid pKSV7-Δ*hly* was transformed into LM928 competent cells by electroporation. Because pKSV7 is a temperature-sensitive shuttle vector, different incubation temperatures were employed during mutant construction to facilitate plasmid maintenance and homologous recombination. Transformants were cultured on chloramphenicol-supplemented BHI agar (10 μg/mL) at 30 °C for 36–48 h and screened by PCR. Positive transformants were cultured in chloramphenicol-containing BHI broth at 42 °C to induce homologous recombination. PCR screening using the vΔ*hly* primer pair was performed every five passages until only the expected 1477-bp amplicon was detected. The verified integrants were subsequently serially passaged in antibiotic-free BHI broth at 30 °C to facilitate the second homologous recombination event. PCR verification was conducted every five passages, and no reversion was observed, as only the 1477-bp amplicon was detected throughout the passaging process. In parallel, cultures were streaked onto BHI agar plates containing chloramphenicol every five passages, and chloramphenicol-sensitive colonies were selected. Candidate mutants were further confirmed by PCR and DNA sequencing. The correctly constructed *hly* deletion mutant was designated LM928Δ*hly*. To evaluate genetic stability, the mutant strain was continuously passaged for 10 generations in antibiotic-free medium and verified by PCR.

#### Construction of the ΔLIPI-4/*hly* double-deletion mutant

To construct the LM928ΔLIPI-4/*hly* double-deletion mutant, the laboratory-preserved LM928ΔLIPI-4 strain was used as the parental strain for *hly* deletion. The *hly* gene was deleted using the same pKSV7-mediated allelic exchange strategy described above, including electroporation, temperature-dependent homologous recombination, and antibiotic-based screening procedures under the same culture conditions. Candidate mutants were verified by PCR and sequencing, and the correctly constructed double-deletion mutant was designated LM928ΔLIPI-4/*hly*.

#### Construction of the *hly* complemented strains

The *hly* gene fragment was PCR-amplified from LM928 genomic DNA using primers *hly*-F and *hly*-R. Both the amplified fragment and the integrative vector pIMK2 were double-digested with PstI and XhoI, purified, and ligated using DNA ligase to generate the recombinant integrative plasmid pIMK2-*hly*. The recombinant plasmid pIMK2-*hly* was introduced into competent cells of LM928Δ*hly* and LM928ΔLIPI-4/Δ*hly* by electroporation. Complemented strains were screened by PCR and confirmed by sequencing, and were designated LM928Δ*hly*::*hly* and LM928ΔLIPI-4/Δ*hly*::*hly*, respectively.

### Western blot analysis of deletion and complemented strains

Total bacterial and secreted proteins were extracted based on previously published methods with minor modifications (Halbedel et al., 2014; Fang et al., 2024).

Strains LM928, LM928Δ*hly*, LM928ΔLIPI-4/Δ*hly*, LM928ΔLIPI-4, LM928Δ*hly*::*hly* and LM928ΔLIPI-4/Δ*hly*::*hly* were streaked onto BHI agar plates for single-colony isolation. Single colonies were inoculated into 50 mL of BHI broth and incubated at 37 °C with agitation at 160 rpm. Bacterial cultures were harvested at the mid-logarithmic growth phase (OD600 ≈ 0.6) by centrifugation at 4 °C. For total bacterial protein extraction, the bacterial pellets were washed twice with sterile PBS and resuspended in a Listeria lysis buffer containing 20 mM Tris-HCl, 150 mM NaCl, 1 mM EDTA, 0.1% SDS, 1% Triton X-100, and 1 mM PMSF. After thorough mixing, the lysates were centrifuged at 12,000 rpm for 20 min at 4 °C to remove unlysed cells and cellular debris. The resulting supernatants were collected as total bacterial protein extracts. Protein concentrations were determined using a BCA Protein Assay Kit, and equal amounts of protein were used for SDS-PAGE and Western blot analysis. For secreted protein extraction, the culture supernatants obtained after bacterial centrifugation were collected and passed through 0.22 μm filters to remove residual bacterial cells. Proteins in the supernatants were precipitated by the addition of 10% trichloroacetic acid (TCA) and incubated on ice overnight. The precipitated proteins were collected by centrifugation, resuspended in Loading Buffer, and used as secreted protein samples. Protein concentrations were determined using a BCA Protein Assay Kit, and equal amounts of protein were loaded for SDS-PAGE and Western blot analysis.

Both total bacterial proteins and secreted proteins were quantified using a BCA protein assay, and equal amounts of protein (20 μg per lane) were loaded for SDS-PAGE. The protein samples were resolved by SDS-PAGE and transferred onto PVDF membranes using a semi-dry transfer system. Secreted proteins were resolved by SDS-PAGE and transferred onto PVDF membranes using a semi-dry transfer system. Membranes were washed three times with TBST (15 min each) and then blocked overnight at 4 °C in TBST containing 5% (w/v) non-fat milk. After blocking, membranes were incubated with anti-LLO primary antibody at a dilution of 1:1000 at 37 °C for 2 h. Following incubation, the membranes were washed three times with TBST (15 min each). Subsequently, membranes were incubated with a horseradish peroxidase (HRP)-conjugated rabbit secondary antibody at a dilution of 1:5000 at 37 °C for 1.5 h, followed by three additional washes with TBST. Protein signals were detected using an enhanced chemiluminescence (ECL) substrate and imaged using a chemiluminescence imaging system.

### Determination of bacterial growth curves

Strains LM928, LM928Δ*hly*, LM928ΔLIPI-4/*hly*, LM928ΔLIPI-4, and LM928Δ*hly*::*hly* were streaked onto BHI agar plates for single-colony isolation. Single colonies were inoculated into 50 mL of BHI broth and incubated at 37 °C with agitation at 160 rpm. The optical density at 600 nm (OD600) was measured every 2 h using three independent cultures for each strain. Growth curves were generated by plotting OD600 values against incubation time to evaluate the effects of *hly* deletion on the growth characteristics of *L. monocytogenes*.

### Determination of hemolytic activity

Strains LM928, LM928Δ*hly*, LM928ΔLIPI-4/*hly*, LM928ΔLIPI-4, and LM928Δ*hly*::*hly* were streaked onto BHI agar plates for single-colony isolation. Single colonies were inoculated into 50 mL of BHI broth and incubated at 37 °C with agitation at 160 rpm, and culture supernatants were obtained by centrifugation at 8,000 rpm for 5 min. A 1% suspension of sheep red blood cells was prepared, and 100 μL of the suspension was prepared, and 100 μL of the suspension was dispensed into each well of a 96-well plate. The bacterial supernatants were serially twofold diluted and mixed with the erythrocyte suspension, with BHI broth used as a negative control. The microplates were incubated statically at 37 °C for 2 h in a bacterial incubator. After incubation, 100 μL of supernatant from each well (carefully avoiding the pellet) was collected, and the absorbance was measured at 550 nm using a microplate reader. The recorded OD550 values were statistically analyzed using one-way ANOVA with SPSS software to assess the hemolytic activity among different *L. monocytogenes* strains.

### RT-qPCR analysis of *hly* gene expression

The six *L. monocytogenes* strains were cultured in brain heart infusion (BHI) broth at 37 °C to the logarithmic growth phase (OD600 ≈ 0.6). Bacterial cells were harvested by centrifugation at 8,000 rpm for 10 min at 4 °C, and the cell pellets were collected after removal of the supernatant. The pellets were ground into a fine powder in liquid nitrogen, and total RNA was extracted using a bacterial RNA extraction kit according to the manufacturer's instructions. Complementary DNA (cDNA) was synthesized by reverse transcription and used as the template for real-time quantitative PCR (RT-qPCR). The transcriptional levels of the *hly* and gyrB genes were determined by RT-qPCR, with gyrB serving as the internal reference gene. The relative expression level of the target gene was calculated using the 2^−ΔΔCt^ method. Each sample was analyzed in three independent biological replicates, with three technical replicates for each biological replicate.

### *In vitro* infection assays

#### Bacterial adhesion, invasion, and intracellular proliferation assays

Strains LM928, LM928Δ*hly*, LM928ΔLIPI-4/Δ*hly*, LM928ΔLIPI-4, LM928Δ*hly*::*hly* and LM928ΔLIPI-4/Δ*hly*::*hly* were streaked onto BHI agar plates for single-colony isolation. Single colonies were inoculated into 50 mL of BHI broth and incubated at 37 °C with agitation at 160 rpm. HCMECs and HTR-8/SV neo cells were seeded into 24-well plates and cultured in RPMI-1640 supplemented with 10% fetal bovine serum (FBS) (complete medium) at 37 °C in a humidified incubator with 5% CO_2_ until they reached approximately 80% confluence (≈2 × 105 cells per well). For the adhesion assay, bacterial cultures were harvested at the mid-logarithmic growth phase (OD600 ≈ 0.6) and used to infect cells at a multiplicity of infection (MOI) of 10. After 1 h of incubation, cells were washed twice with PBS to remove non-adherent bacteria. Subsequently, the cells were lysed with 1 mL of 0.2% Triton X-100 for 5 min, and lysates were collected, serially diluted, and plated onto BHI agar plates. Plates were incubated at 37 °C for 24 h, and colonies were counted to determine colony-forming units (CFUs), which represent adherent bacteria.

To quantify bacterial invasion, infected cells were incubated in complete medium containing gentamicin (100 μg/mL) for 1 h, and this time point was designated as 0 h post-invasion. This assay was designed to evaluate bacterial entry efficiency and early intracellular survival following host cell invasion. Cells were washed twice with 1 × PBS and lysed with 1 mL of 0.2% Triton X-100 for 1 min. Lysates were collected into 2 mL microtubes, serially diluted, plated on BHI agar, and incubated at 37 °C for 24 h. Intracellular bacterial numbers were determined by colony counting and expressed as CFUs. For intracellular proliferation assays, infected cells were maintained in medium containing gentamicin (10 μg/mL).This assay was used to assess the ability of intracellular bacteria to survive and proliferate within host cells over time. At 4, 8, and 12 h post-infection, cells were washed with PBS to remove extracellular bacteria and lysed with 0.2% Triton X-100. Lysates were serially diluted and plated onto BHI agar, followed by incubation at 37 °C for 12 h. CFUs were determined at each time point to evaluate intracellular replication of *L. monocytogenes*.

#### Analysis of adhesion, invasion, and intracellular proliferation data

Adhesion and invasion rates from all strains (wild-type, single-deletion mutants, and the double-deletion mutant) were compiled into a single dataset and normalized to the wild-type control. For intracellular proliferation, CFU counts were collected at each time point and log10-transformed prior to analysis. To test whether deletion of *hly* and LIPI-4 produced an interaction effect, we calculated the expected phenotype of the double mutant under an independent-action model using E_expected = (E_gene1 × E_gene2) / E_WT, where E_gene1 and E_gene2 represent the effect values of the two single mutants (adhesion rate, invasion rate, or log10 CFU) and EW_T denotes the corresponding wild-type value. A synergistic interaction was inferred when the observed double-mutant phenotype was significantly lower than the expected value, whereas similar observed and expected values were interpreted as independent effects and higher-than-expected values were considered indicative of antagonism.

### Mouse virulence assay

Six-week-old female ICR mice were randomly assigned to six groups, with six mice per experimental group and three mice in the control group. All mice were acclimatized for 1 week under standard laboratory conditions prior to infection. Intraperitoneal injection was selected as a standard and highly reproducible route for establishing systemic *L. monocytogenes* infection, enabling controlled bacterial dissemination into the bloodstream and subsequent colonization of target organs. Compared with oral infection, this route provides greater experimental consistency and reduces variability in bacterial uptake. Wild-type and mutant *L. monocytogenes* strains were inoculated into 5 mL of BHI broth and cultured overnight at 37 °C with shaking. Bacterial cells were harvested by centrifugation, washed twice with sterile PBS, and resuspended to a final concentration of 1 × 107 CFU/mL. Mice in the experimental groups were intraperitoneally injected with 0.1 mL of the bacterial suspension (1 × 106 CFU), whereas control mice received 0.1 mL sterile PBS. At 72 h post-infection, mice were humanely euthanized by cervical dislocation following physical restraint. The spleen, liver, and brain were aseptically collected into 2 mL tubes containing sterile grinding beads and 1 mL of sterile phosphate-buffered saline (PBS). Tissues were homogenized, serially diluted, and plated on BHI agar for CFU enumeration to determine bacterial burdens in infected organs. Based on the systemic infection pathway of *L. monocytogenes*, intraperitoneal inoculation leads to hematogenous dissemination and subsequent colonization of major reticuloendothelial organs, particularly the spleen and liver, while the bacterium is also capable of crossing the blood-brain barrier to invade the central nervous system. Therefore, spleen, liver, and brain were selected to assess systemic bacterial burden and neuroinvasion.

### Ethics statement

All animal experiments were conducted in accordance with the National Guidelines for Housing and Care of Laboratory Animals (China) and approved by the Biology Ethics Committee of Shihezi University (Approval No. A2021-26; Approval date: 10 March 2021), as well as the AVMA Guidelines for the Euthanasia of Animals (2020).

### Data analysis and statistical evaluation

Data were analyzed using SPSS software. Differences among groups were evaluated statistically, and results were annotated with lowercase letters, where groups sharing the same letter were not significantly different (*p* > 0.05), whereas different letters indicated statistically significant differences (*p* < 0.05).

## Results

### Successful construction of mutant and complemented strains

All single-deletion, double-deletion, and complemented strains were successfully constructed and verified by PCR, DNA sequencing, and restriction enzyme digestion (Supplementary Figure 1).

### Western blot verification of LLO expression in mutant and complemented strains

**Figure 1:**
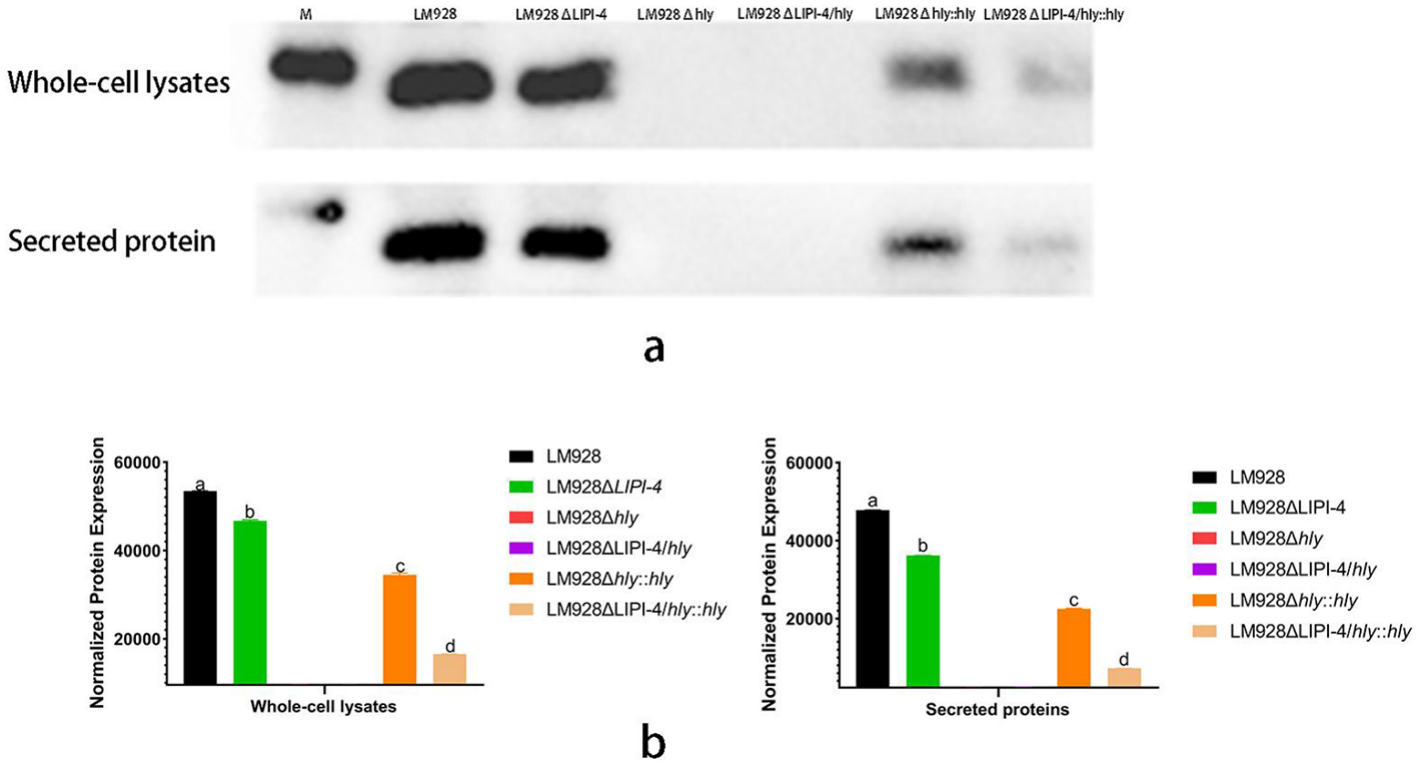
**(a)** Western blot analysis of LLO expression in six *L. monocytogenes* strains. The predicted molecular weight of the LLO protein is 56 kDa. **(b)** Densitometric analysis of LLO protein expression based on Western blot results. Band intensities were quantified using ImageJ software and normalized to the wild-type strain. Data are presented as mean ± SD from three independent experiments.

Western blot analysis was performed using rabbit anti-LLO antibody as the primary antibody. Total and secreted proteins extracted from LM928, LM928Δ*hly*, LM928ΔLIPI-4, LM928ΔLIPI-4/*hly*, LM928Δ*hly*::*hly* and LM928ΔLIPI-4/*hly*::*hly* strains were examined. LLO protein was detected in LM928, LM928ΔLIPI-4, and the complemented strain LM928Δ*hly*::*hly*, whereas no LLO expression was observed in LM928Δ*hly* and LM928ΔLIPI-4/*hly* (Figure 1a). As shown in Figure 1b, LLO expression was significantly higher in LM928 than in LM928ΔLIPI-4, which in turn exhibited significantly higher expression than the complemented strain LM928Δ*hly*::*hly*. The LM928ΔLIPI-4/Δ*hly*::*hly* strain showed the lowest LLO expression level among the tested strains (*P* < 0.05).

### Growth curve analysis of different *L. monocytogenes* strains

**Figure 2:**
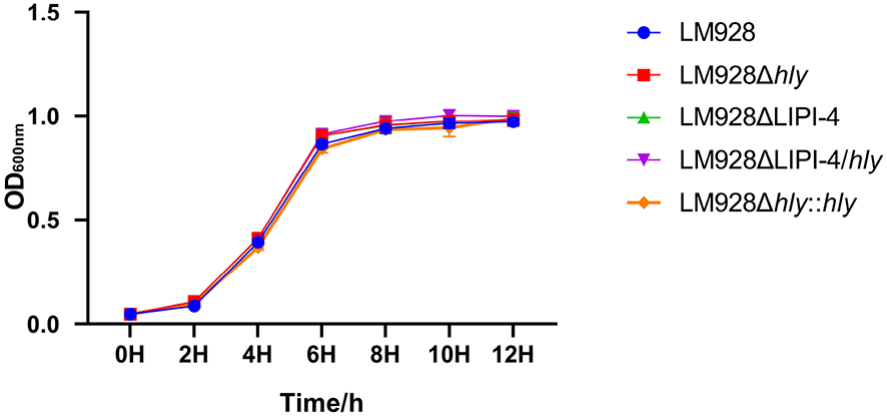
Growth curves of the strains at 37 °C.

The strains LM928, LM928Δ*hly*, LM928ΔLIPI-4, LM928ΔLIPI-4/*hly*, LM928Δ*hly*::*hly*, and LM928ΔLIPI-4/*hly*::*hly* were cultured in BHI broth at 37 °C with shaking at 160 rpm. Growth was monitored by measuring OD600 every 2 h over a 12 h period. All strains exhibited a similar growth pattern, including a lag phase (0–2 h), an exponential growth phase (2–8 h), and a stationary phase (8–12 h). No significant differences in growth kinetics were observed among the strains (Figure 2), indicating that deletion of *hly* and LIPI-4 did not affect bacterial growth or viability under the tested conditions.

### Hemolytic activity analysis of different *L. monocytogenes* strains

**Figure 3:**
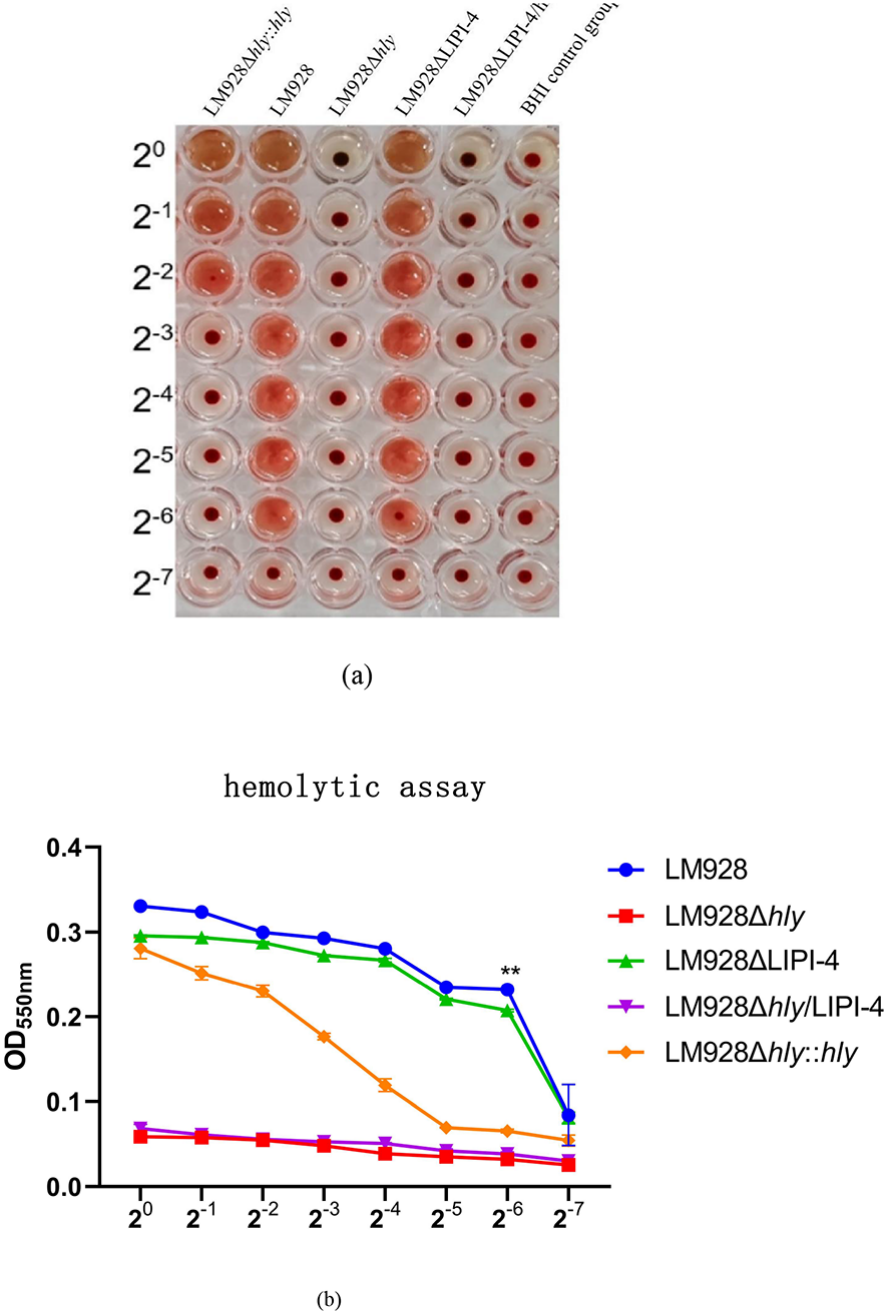
Hemolytic results of *L. monocytogenes* wild-type, mutant, and complemented strains **(a)** Hemolysis activity chart of each strain **(b)** Hemolytic activity curves (OD550) of each strain.

Hemolytic assay results showed that the wild-type LM928 exhibited a hemolytic titer of 1:64, whereas the LIPI-4 deletion mutant (LM928ΔLIPI-4) showed a reduced titer of 1:32. No hemolysis was detected in either the *hly*-deficient strain (LM928Δ*hly*) or the *hly*/LIPI-4 double-deletion mutant (LM928ΔLIPI-4/*hly*) at any dilution. Complementation of *hly* (LM928Δ*hly*::*hly*) partially restored hemolytic activity, with a titer of 1:4 (Figure 3). Statistical analysis further confirmed that the hemolytic titer of the LIPI-4 deletion mutant was significantly reduced compared with the wild-type strain (1:64 vs. 1:32, *p* < 0.05).

### RT-qPCR analysis of *hly* gene expression

**Figure 4:**
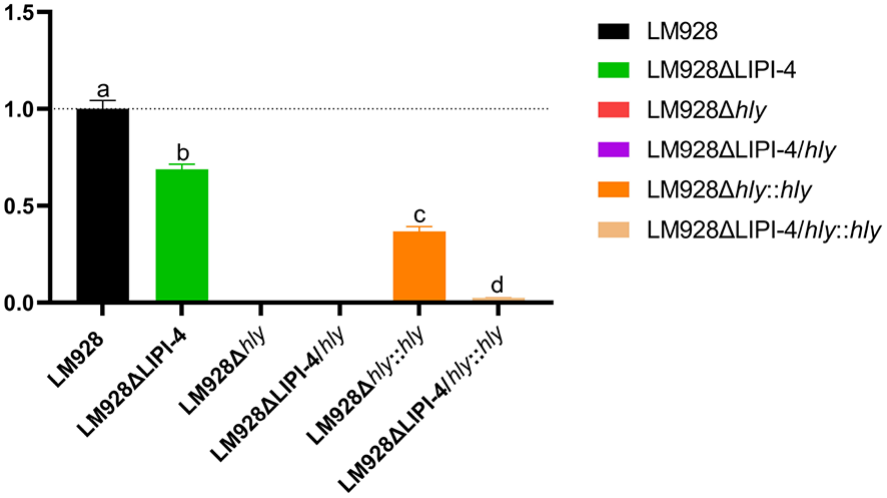
Relative transcription levels of *hly* in different *L. monocytogenes* strains determined by RT-qPCR. Total bacterial RNA was extracted and reverse-transcribed into cDNA for quantitative analysis. Relative expression levels were normalized to the housekeeping gene gyrB. Data are presented as mean ± SD from three independent experiments. Different letters indicate statistically significant differences between groups (*P* < 0.05).

To further investigate whether the differences in LLO protein expression among the strains were associated with changes in *hly* transcription, the relative expression of the *hly* gene was determined by RT-qPCR. The results showed that no *hly* expression was detected in the LM928Δ*hly* and LM928ΔLIPI-4/*hly* strains. Among the remaining strains, the wild-type strain LM928 exhibited the highest *hly* transcription level, which was significantly higher than that of LM928ΔLIPI-4. In addition, the *hly* expression level in LM928ΔLIPI-4 was significantly higher than that in the complemented strain LM928Δ*hly*::*hly*, whereas LM928Δ*hly*::*hly* showed significantly higher *hly* expression than LM928ΔLIPI-4/*hly*::*hly* (*P* < 0.05) (Figure 4).

### Adhesion, invasion, and intracellular proliferation of five *L. monocytogenes* strains in different host cells

**Figure 5:**
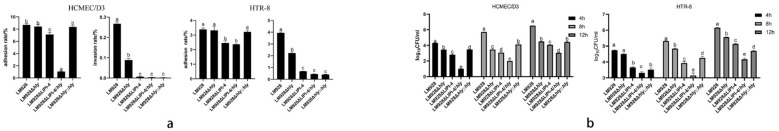
**(a)** Adhesion and invasion rates of *L. monocytogenes* strains in HCMEC/D3 and HTR-8 cells. Bars labeled with different letters represent statistically significant differences (p < 0.05), as determined by one-way ANOVA. **(b)** Intracellular proliferation of *L. monocytogenes* strains in HCMEC/D3 and HTR-8 cells.

Human cerebral microvascular endothelial cells (HCMEC/D3) and placental trophoblast cells (HTR-8) were used to assess adhesion and invasion of different *L. monocytogenes* strains *in vitro*. Cells were infected with LM928, LM928Δ*hly*, LM928ΔLIPI-4, LM928ΔLIPI-4/*hly*, and LM928Δ*hly*::*hly*, and bacterial adhesion, invasion, and intracellular replication were quantified by CFU enumeration at the indicated time points. In HCMEC/D3 and HTR-8 cells, deletion of *hly* did not significantly affect adhesion (*p* > 0.05), whereas deletion of LIPI-4 significantly decreased bacterial attachment compared with the wild-type strain (*p* < 0.01) (Figure 5(a)). Notably, the double mutant LM928ΔLIPI-4/*hly* showed a further reduction in adhesion in HCMEC/D3 cells relative to both the wild-type strain and the LM928ΔLIPI-4 single mutant, consistent with a potential interaction between *hly* and LIPI-4. In invasion assays, both LM928Δ*hly* and LM928ΔLIPI-4 displayed significantly reduced invasion efficiencies compared with LM928 (*p* < 0.01). In addition, LM928Δ*hly* invaded at a higher level than LM928ΔLIPI-4 (*p* < 0.01), supporting a stronger contribution of LIPI-4 to cellular invasion.

### Intracellular proliferation of five *L. monocytogenes* strains in HCMEC/D3 and HTR-8 cells

As a facultative intracellular pathogen, *L. monocytogenes* relies heavily on its capacity for intracellular replication to establish infection and disseminate within host tissues. As shown in Figure 5(b), all strains demonstrated time-dependent intracellular proliferation in both cell types. Nevertheless, bacterial replication of all mutant strains was consistently lower in HCMEC/D3 cells compared with HTR-8 cells, suggesting a reduced ability to proliferate within brain endothelial cells. In HCMEC/D3 cells, LM928 displayed significantly higher intracellular proliferation compared with LM928Δ*hly*, LM928ΔLIPI-4, and the double mutant LM928ΔLIPI-4/*hly* at all time points (4 h, 8 h, and 12 h; *p* < 0.01). However, no significant difference (*p* > 0.05) was observed between LM928 and the complemented strain LM928Δ*hly*::*hly*, indicating successful restoration of *hly*-associated function. In contrast, within HTR-8 cells, no significant difference (*p* > 0.05) was observed between LM928 and LM928Δ*hly* at 4 h. By 8 h and 12 h, however, intracellular CFU counts followed the order LM928 > LM928Δ*hly* > LM928ΔLIPI-4 > LM928ΔLIPI-4/*hly*, with statistically significant differences among all groups (*p* < 0.01). These findings indicate that both *hly* and LIPI-4 contribute to efficient intracellular replication, with LIPI-4 playing a more pronounced role in sustaining bacterial proliferation.

### Bacterial loads of Five *L. monocytogenes* strains in mouse brain, liver and spleen tissues

**Figure 6:**
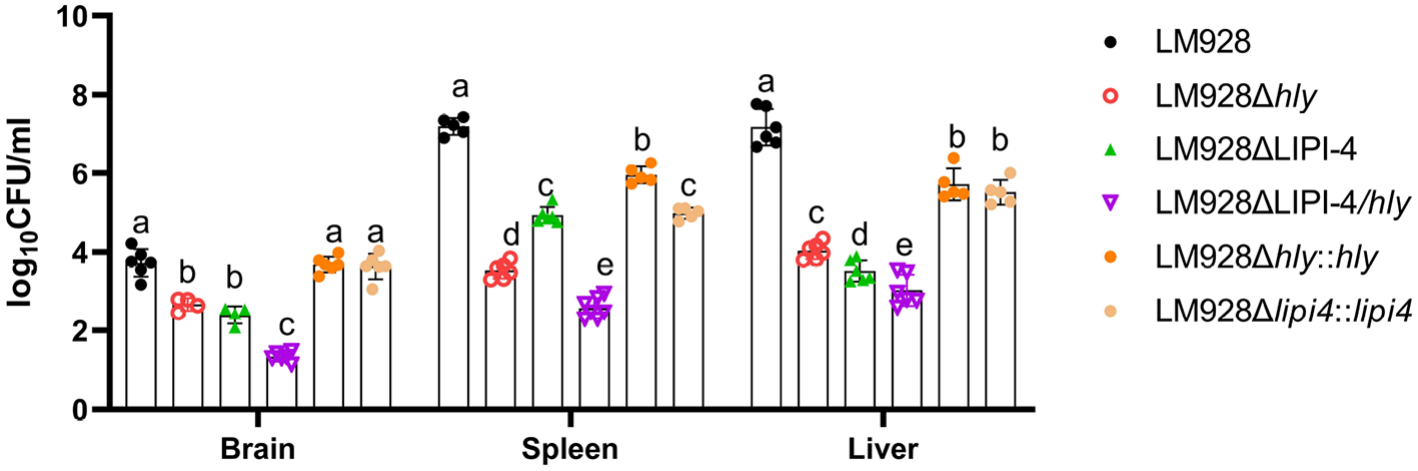
Bacterial loads of *L. monocytogenes* wild-type, mutant, and complemented strains in mouse tissues and organs.

At 72 h post-infection, mouse brain and spleen tissues were homogenized, serially diluted, and plated for CFU enumeration (Figure 6). In the brain, bacterial loads of LM928, LM928ΔLIPI-4::LIPI-4, and LM928Δ*hly*::*hly* showed no significant differences (*p* > 0.05). LM928Δ*hly* and LM928ΔLIPI-4 also displayed comparable bacterial burdens; however, overall bacterial counts followed a descending trend of LM928 > LM928Δ*hly* > LM928ΔLIPI-4/*hly*, with statistically significant differences among groups. In the spleen, bacterial loads of LM928ΔLIPI-4 and LM928ΔLIPI-4::LIPI-4 were not significantly different (*p* > 0.05), whereas all other strains exhibited significant differences (*p* < 0.01). The bacterial burden ranked as follows: LM928 > LM928Δ*hly*::*hly* > LM928ΔLIPI-4 > LM928Δ*hly* > LM928ΔLIPI-4/*hly*, indicating that deletion of *hly* and LIPI-4 markedly attenuated *L. monocytogenes* virulence *in vivo*.

## Discussion

*L. monocytogenes* is a major foodborne pathogen responsible for severe invasive diseases, including meningitis, sepsis, and fetal infections, with mortality rates of approximately 20–30% (Jashari et al., 2024; Costa et al., 2024; Żurawik et al., 2024). Its pathogenicity relies on a coordinated virulence network in which the classical pathogenicity island LIPI-1 and the lineage-associated LIPI-4 play distinct but potentially interconnected roles. While LIPI-1 encodes core virulence determinants such as (LLO, *hly*) and ActA that mediate intracellular survival and cell-to-cell spread (Wiktorczyk-Kapischke et al., 2023a), LIPI-4 is primarily associated with metabolic adaptation and enhanced neuro- and placental tropism (Xia et al., 2025). However, how metabolic regulatory modules interface with classical virulence systems remains incompletely understood. Importantly, this study identifies a previously uncharacterized functional link between the LIPI-4 metabolic island and the virulence factor *hly*/LLO. Our results show that LIPI-4 not only contributes to metabolic adaptation but also influences *hly* expression and LLO function, indicating a regulatory role beyond its established metabolic function. Notably, LIPI-4 deletion caused stronger phenotypic defects than *hly* deletion in several assays, suggesting a hierarchical relationship between metabolic adaptation and virulence execution. In addition, complementation data indicate that LIPI-4-dependent metabolic context affects *hly* restoration efficiency, revealing a context-dependent regulatory mechanism in *L. monocytogenes*.

In this study, we demonstrate that LIPI-4 significantly contributes to adhesion, invasion, intracellular replication, and *in vivo* dissemination. Notably, the LIPI-4 deletion mutant exhibited more pronounced defects than the *hly* mutant in several infection-related assays, and the double mutant showed the most severe attenuation, supporting a cooperative contribution of LIPI-4 and *hly* to full virulence. These findings suggest that LIPI-4 influences host interaction processes beyond classical metabolic transport functions. Consistent with this observation, previous studies have shown that LIPI-4-positive CC4 strains are overrepresented in central nervous system and pregnancy-associated infections, and deletion of the LIPI-4-associated PTS cluster significantly impairs blood-brain barrier traversal, highlighting its role in tissue tropism rather than direct cytolytic activity (Maury et al., 2016).

Mechanistically, RT-qPCR, Western blotting, and hemolysis assays revealed that deletion of LIPI-4 leads to reduced *hly* transcription, decreased LLO protein abundance, and attenuated hemolytic activity. Importantly, complementation of *hly* in the LIPI-4-deficient background failed to fully restore LLO expression to wild-type levels, indicating that LIPI-4 is required to maintain an optimal intracellular regulatory context for *hly* expression. Previous work has similarly demonstrated that LLO expression is highly sensitive to metabolic status, where carbon availability can modulate PrfA activity and downstream *hly* transcription without alterations in virulence gene content, emphasizing the metabolic dependency of LLO regulation (Johansson and Freitag, 2019).

To explain this functional coupling, we propose a multi-level model linking metabolic adaptation, global regulation, and virulence execution. LIPI-4 encodes a cellobiose-family phosphotransferase system (PTS) that participates in carbohydrate uptake and metabolic adaptation, thereby influencing intracellular carbon flux under host conditions. These metabolic signals are integrated into the PrfA-centered regulatory network, which acts as a central hub connecting environmental and metabolic cues to virulence gene expression. PrfA-dependent regulation ultimately controls *hly* transcription and LLO production, thereby governing phagosomal escape and intracellular survival. In agreement with this model, previous studies have shown that PTS-mediated phosphorylation states and carbon catabolite repression systems regulate PrfA activation through intracellular metabolite ratios such as PEP/pyruvate (Sonnleitner, 2025), directly linking metabolic sensing to virulence regulation (Meireles et al., 2024).

Taken together, our findings support a model in which LIPI-4 functions as a metabolic-regulatory interface that indirectly modulates LLO-dependent virulence through global regulatory networks rather than acting as a classical virulence determinant. In this context, LIPI-4 fine-tunes the activity of LIPI-1-encoded effector systems, highlighting an interaction between lineage-specific metabolic modules and conserved virulence machinery. More broadly, similar hierarchical organization between conserved virulence islands and accessory metabolic modules has been reported in other intracellular pathogens, suggesting that metabolic rewiring represents a common evolutionary strategy for optimizing host adaptation (Acierno et al., 2025).

From an evolutionary perspective, LIPI-1 represents a conserved and essential virulence island encoding core effectors required for intracellular infection, whereas LIPI-4 is primarily restricted to specific lineages (notably CC4) and is associated with host adaptation and tissue tropism. This distinction supports the notion that LIPI-4 contributes to strain-specific modulation of virulence rather than universal pathogenic mechanisms. Accordingly, the regulatory interaction observed here may not be generalizable across all *L. monocytogenes* lineages. Consistently, comparative genomic studies have shown that LIPI-4 is enriched in hypervirulent CC4 isolates associated with central nervous system and pregnancy-related listeriosis, while being absent from most other lineages (Zakrzewski et al., 2023; Lee et al., 2023; Maury et al., 2019).

*In vivo* experiments using an ICR mouse model further confirmed the cooperative contribution of LIPI-4 and *hly* to systemic infection. Following intraperitoneal infection, bacteria disseminated via the bloodstream and colonized primarily the spleen and liver, with subsequent invasion of the brain. Deletion of either gene significantly reduced bacterial burden in these organs, with the double mutant showing the most severe attenuation, consistent with cooperative roles in systemic dissemination and neuroinvasion. However, this model bypasses the natural oral infection route and does not fully recapitulate human-specific tissue environments such as placenta and brain microarchitecture, which are key targets of LIPI-4-associated infection.The route of infection has been demonstrated to significantly affect tissue tropism and disease progression in *L. monocytogenes* (D'orazio, 2014).

An additional limitation of this study is the use of a single clinical isolate (LM928), which may not fully represent the genetic diversity of *L. monocytogenes* lineages. Another limitation of the present study is that the pIMK2-based complementation system did not fully restore the phenotypes associated with *hly* deletion. Although *hly* complementation partially recovered LLO production, the complemented strain failed to restore several virulence-associated phenotypes to wild-type levels and, unexpectedly, exhibited reduced invasion efficiency compared with the Δ*hly* mutant. Similar limitations of complementation systems have been reported in *Listeria monocytogenes* and related intracellular pathogens (Lucas Stelling et al., 2010). This suggests that plasmid-based complementation may not fully recapitulate the native chromosomal regulatory context of *hly*. The function of LLO is tightly controlled by precise temporal regulation, expression level, and intracellular compartmentalization (Dancz et al., 2002). Dysregulation of *hly* expression outside its native genomic context may therefore alter toxin activity and infection dynamics, resulting in incomplete phenotypic restoration (Glomski and Portnoy, 2003). Importantly, RT-qPCR and Western blot analyses showed that both *hly* transcription and LLO protein levels in the ΔLIPI-4/Δ*hly*::*hly* were consistently lower than those in the Δ*hly*::*hly*. This indicates that LIPI-4 deficiency influences the efficiency of *hly* restoration under complementation conditions. Nevertheless, these expression differences remain consistent with the overall phenotypic trends observed in this study and do not affect the main conclusion that LIPI-4 is functionally associated with regulation of *hly*/LLO-mediated virulence.

Future studies employing chromosomal complementation, promoter activity analyses, and transcriptomic approaches will be required to further dissect the molecular circuitry linking metabolic adaptation with virulence gene regulation in *L. monocytogenes*.

## Data Availability

The original contributions presented in the study are included in the article/supplementary material, further inquiries can be directed to the corresponding author/s.
