# Supplementary material for: Functional cooperation between LIPI-4 and listeriolysin O governs intracellular replication and systemic virulence of Listeria monocytogenes
Source: Front Microbiol. 2026 Jul 17;17:1853120. doi: 10.3389/fmicb.2026.1853120 (PMC13423915; doi:10.3389/fmicb.2026.1853120)
Supplement: Supplementary file 1 [file Table_1.docx]

Supplementary Table S1. Genetic organization of the LIPI-4 pathogenicity island and the *hly* gene in Listeria monocytogenes LM928.

| **Gene name** | **Locus tag** | **Genomic location (bp)** | **Reference accession** | **Description** |
| --- | --- | --- | --- | --- |
| *hly* | GNG71_RS01085 | 2794868-2796457 | NZ_CP046478.1 | cholesterol-dependent cytolysin listeriolysin O |
| *Lm*4b_02324 | GNG71_RS12385 | 552699-554006 | NZ_CP046478.1 | 6-phospho-alpha-glucosidas |
| *Lm*4b_02325 | GNG71_RS12390 | 550763-552679 | NZ_CP046478.1 | BglG family transcription antiterminator |
| *Lm*4b_02326 | GNG71_RS12395 | 550001..550747 | NZ_CP046478.1 | \| **Carbohydrate deacetylase** \| \| --- \| |
| *Lm*4b_02327 | GNG71_RS12400 | 549,674–549,988 | NZ_CP046478.1 | PTS lactose/cellobiose transporter subunit IIA |
| *Lm*4b_02328 | GNG71_RS12405 | 549318..549674 | NZ_CP046478.1 | PTS sugar transporter subunit IIB |
| *Lm*4b_02329 | GNG71_RS12410 | 547997..549298 | NZ_CP046478.1 | PTS sugar transporter subunit **IIC** |

Note: Gene annotations were retrieved from the NCBI GenBank database based on the complete genome sequence of Listeria monocytogenes LM928 (chromosome accession no. NZ_CP046478.1).
Listeria pathogenicity island 4 (LIPI-4) corresponds to a six-gene cluster originally described in serotype 4b strains (lm4b_02324–lm4b_02329), encoding a phosphoenolpyruvate-dependent phosphotransferase system (PTS). In strain LM928, the homologous genes constituting LIPI-4 are listed in this table using their NCBI locus_tag identifiers.
LIPI-4 represents a pathogenicity island composed of six contiguous genes encoding components of the PTS, as well as associated regulatory and carbohydrate metabolism proteins. Except for *hly*, which has an assigned gene name, the remaining genes do not have official gene symbols and are therefore designated as putative genes.

Supplementary Table S2. Primer sequences used for construction and verification of the *hly* mutant and complemented strains.

| Primers | Sequence (5'-3') | Restriction site | Product length (bp) |
| --- | --- | --- | --- |
| Δ*hly* up-F  Δ*hly* up-R | AACTGCAGTTCTTTATGGGCTTATTCCAG 3‘  TTTTTATTAATTTTTAAAAGGGTTTCACTCTCCTTCTA | *Pst*Ⅰ | 364 |
| Δ*hly* dn-F  Δ*hly* dn-R | TAGAAGGAGAGTGAAACCCTTTTAAAAATTAATAAAAA  CGGGATCCGCCTGAAAAGCTATTACCATG | *Bam*HⅠ | 381 |
| vΔ*hly* A-F  vΔ*hly* A-R | CCGAACTGCATGCCGAATTTG  TCAGAACTTAGCGCGAGCGTA |  | 3067/1477 |
| cΔ*hly*-F  cΔ*hly*-R | AACTGCAGATGAAAAAAATAATGCTAGTT  CCCTCGAGTTATTCGATTGGATTATCTACACTA | *Pst*Ⅰ  *Xho*Ⅰ | 1590 |

Note: The restriction enzyme sites are underlined.


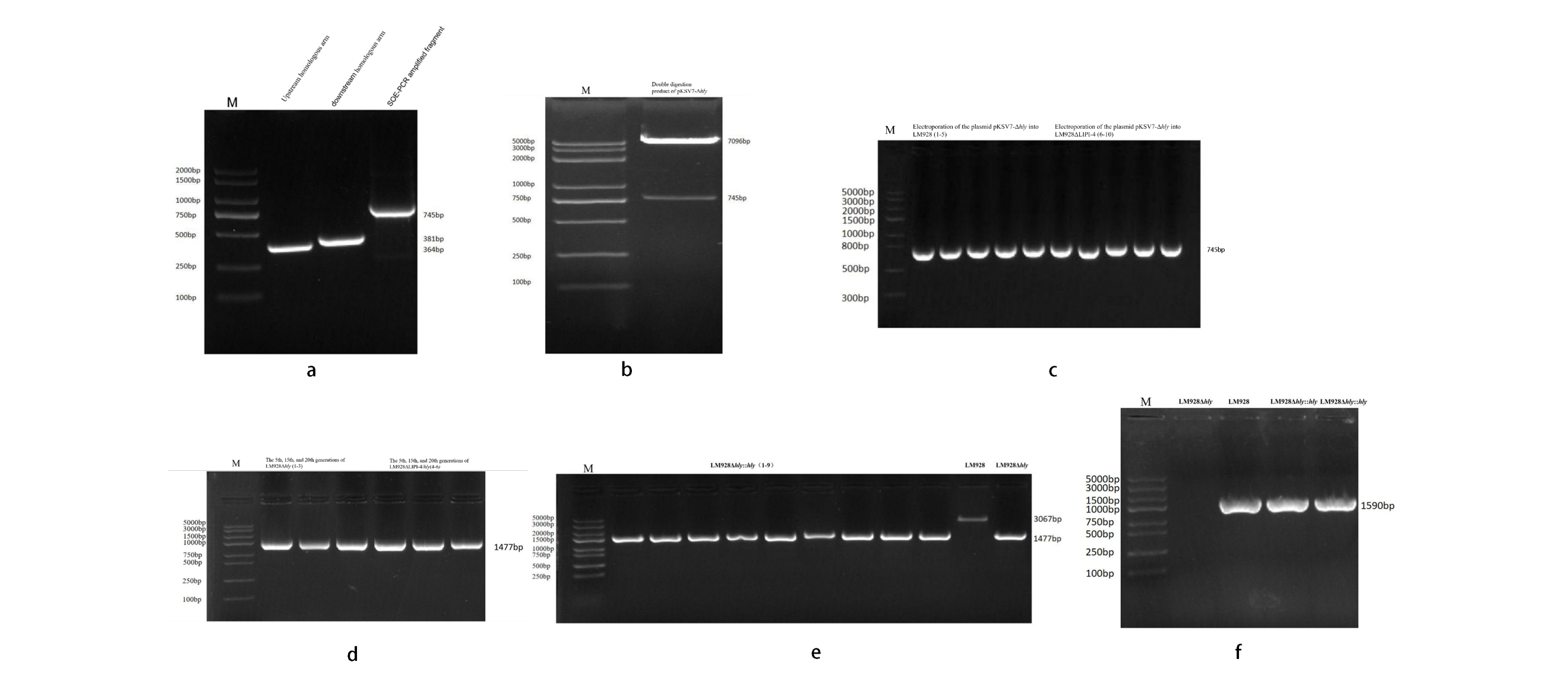


Supplementary Figure S1 **(a)** Amplification and fusion results of the upstream and downstream homologous arms of the *hly* gene by SOE-PCR. **(b)** Identification of the double digestion results of the pKSV7-Δ*hly* plasmid. **(c)**PCR verification of positive clones obtained after electroporation of the recombinant plasmid pKSV7-Δ*hly*. **(d)** PCR verification of the *hly* deletion mutant using vΔ*hly* primers.**(e)** PCR verification of the LM928Δ*hly*::*hly* complemented strain using vΔ*hly* primers.**(f)** PCR verification of the LM928Δ*hly*::*hly* complemented strain using cΔ*hly* primers.


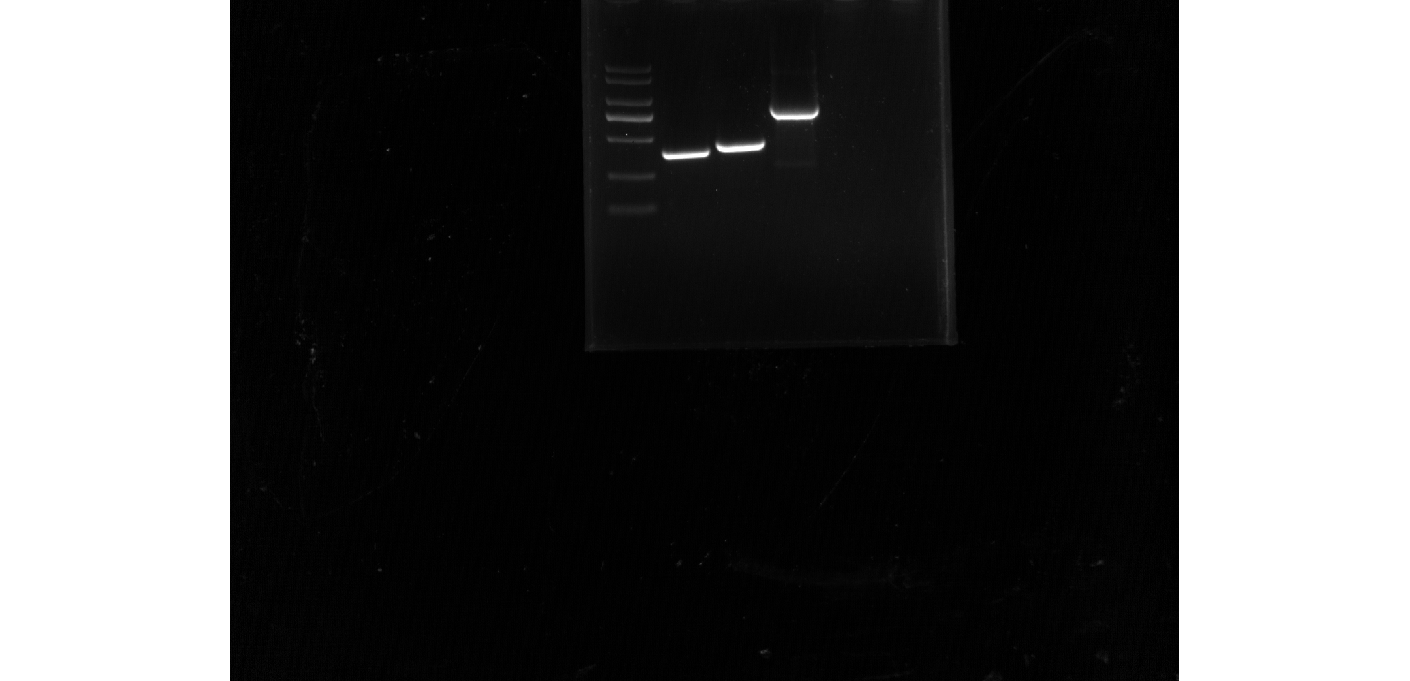


Supplementary Figure S2****.**** Full uncropped original agarose gel images corresponding to Supplementary Figure S1(a).This is the unedited, full-length scan of the agarose gel, showing the complete marker ladder and all loaded samples. It confirms the identity, size, and integrity of the nucleic acid fragments presented in the cropped main figure.


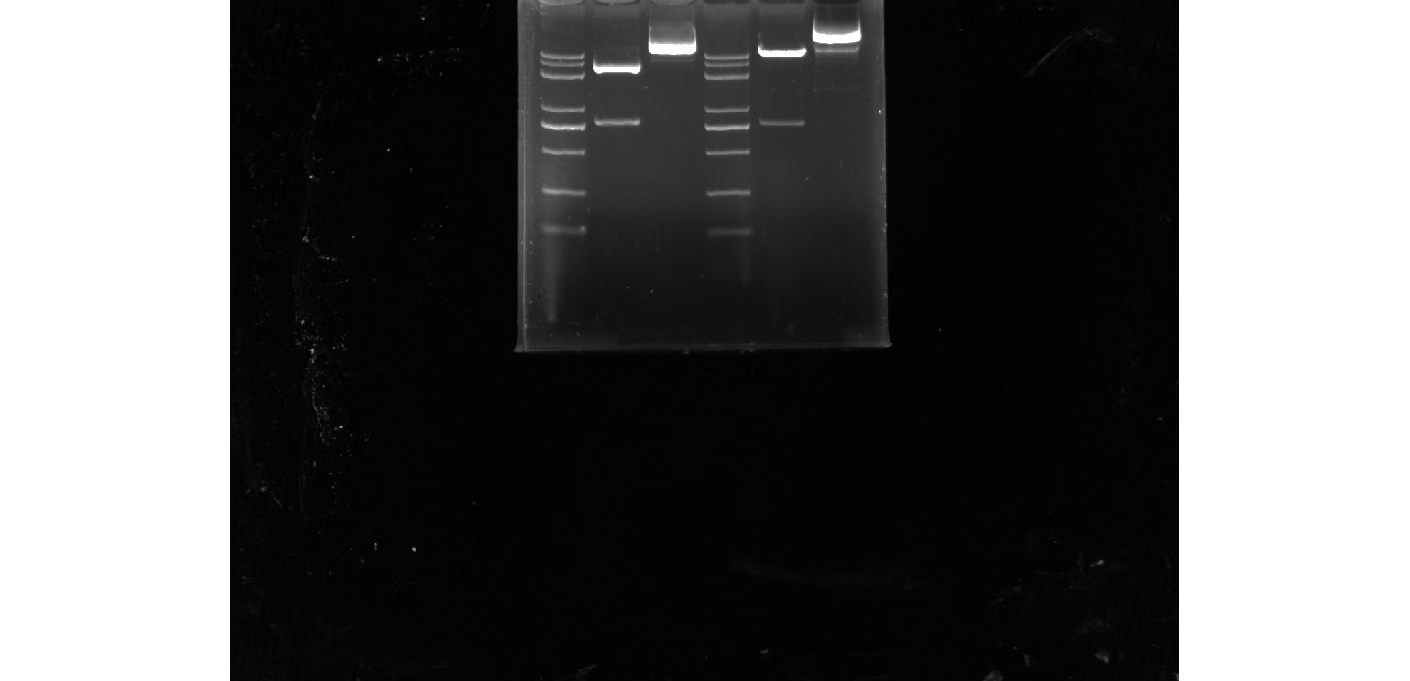


Supplementary Figure S3****.**** Full uncropped original agarose gel images corresponding to Supplementary Figure S1(b). Lane 1 and Lane 4 are the marker ladders. Lane 5 represents the experimental group used in the main manuscript, while lanes 2, 3, 6 correspond to the double enzyme digestion failure groups.These additional bands do not affect the experimental results and conclusions presented in the main manuscript.


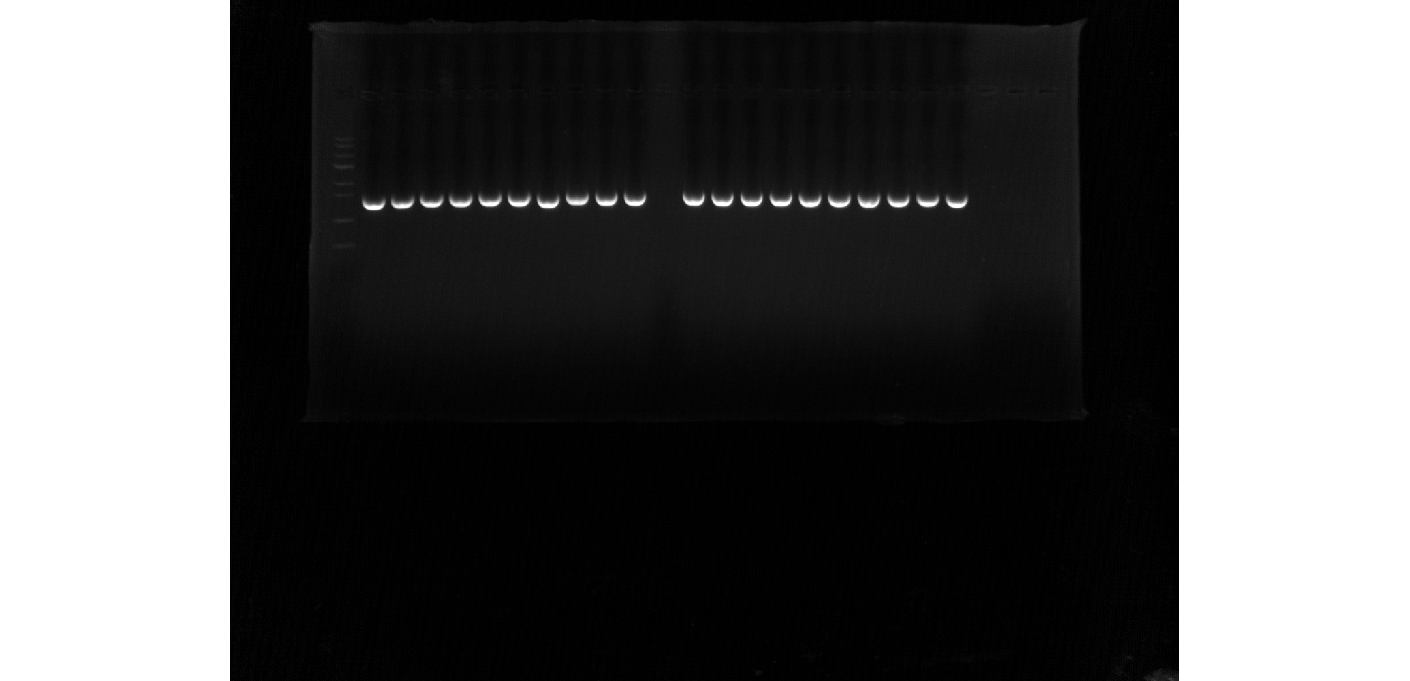


Supplementary Figure S4: Full uncropped original agarose gel images corresponding to Supplementary Figure S1(c). Lane 1 is the marker ladder. Lanes 2 to 11 correspond to the experimental groups presented in the manuscript. Lanes 13 to 22 represent the replicate experimental groups. The additional bands in the replicate groups do not affect the interpretation of the results presented in the main manuscript.


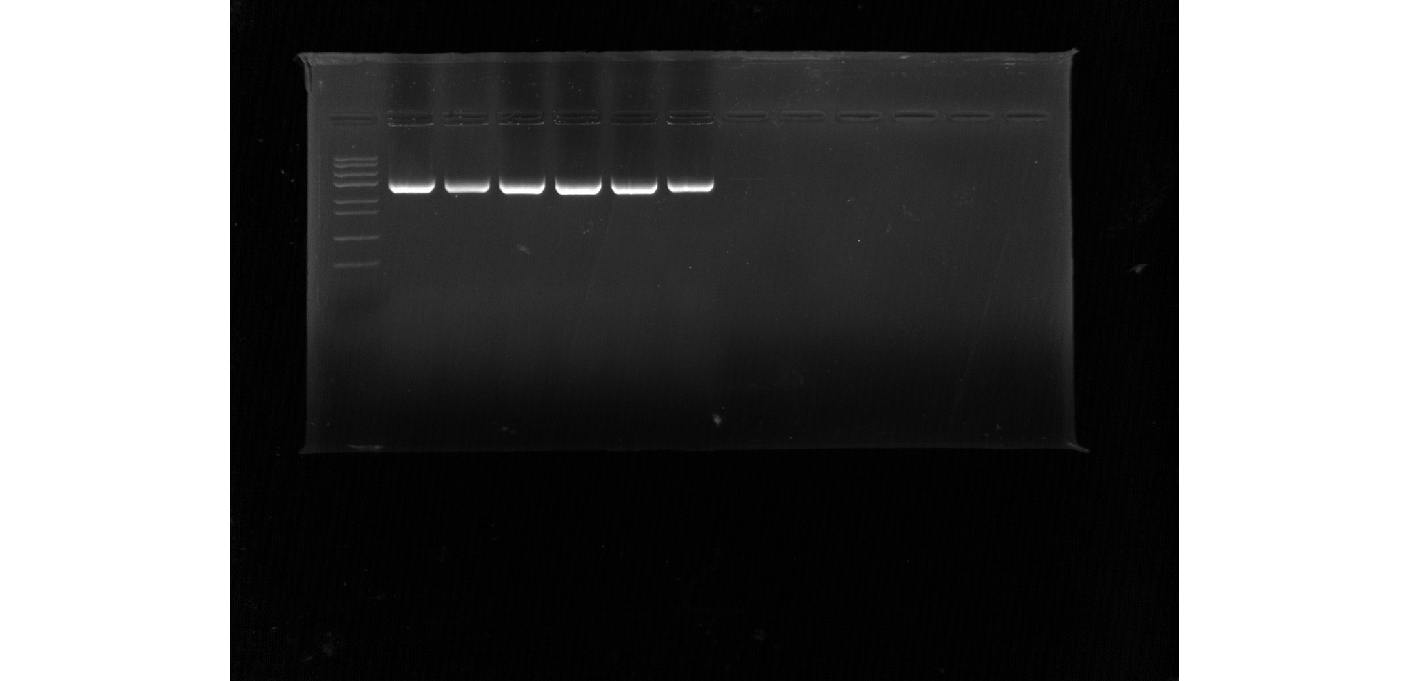


Supplementary Figure S5****.**** Full uncropped original agarose gel images corresponding to Supplementary Figure S1(d).This is the unedited, full-length scan of the agarose gel, showing the complete marker ladder and all loaded samples. It confirms the identity, size, and integrity of the nucleic acid fragments presented in the cropped main figure.


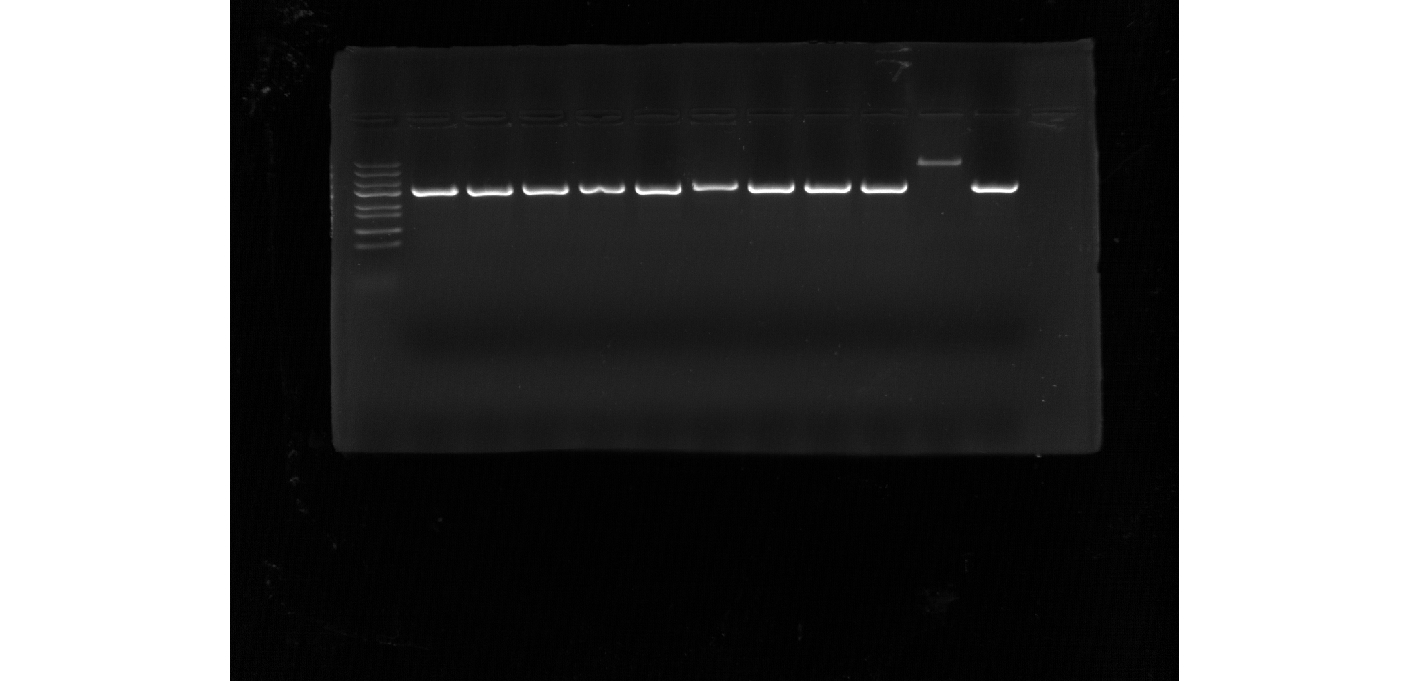


Supplementary Figure S6****.**** Full uncropped original agarose gel images corresponding to Supplementary Figure S1(e).This is the unedited, full-length scan of the agarose gel, showing the complete marker ladder and all loaded samples. It confirms the identity, size, and integrity of the nucleic acid fragments presented in the cropped main figure.


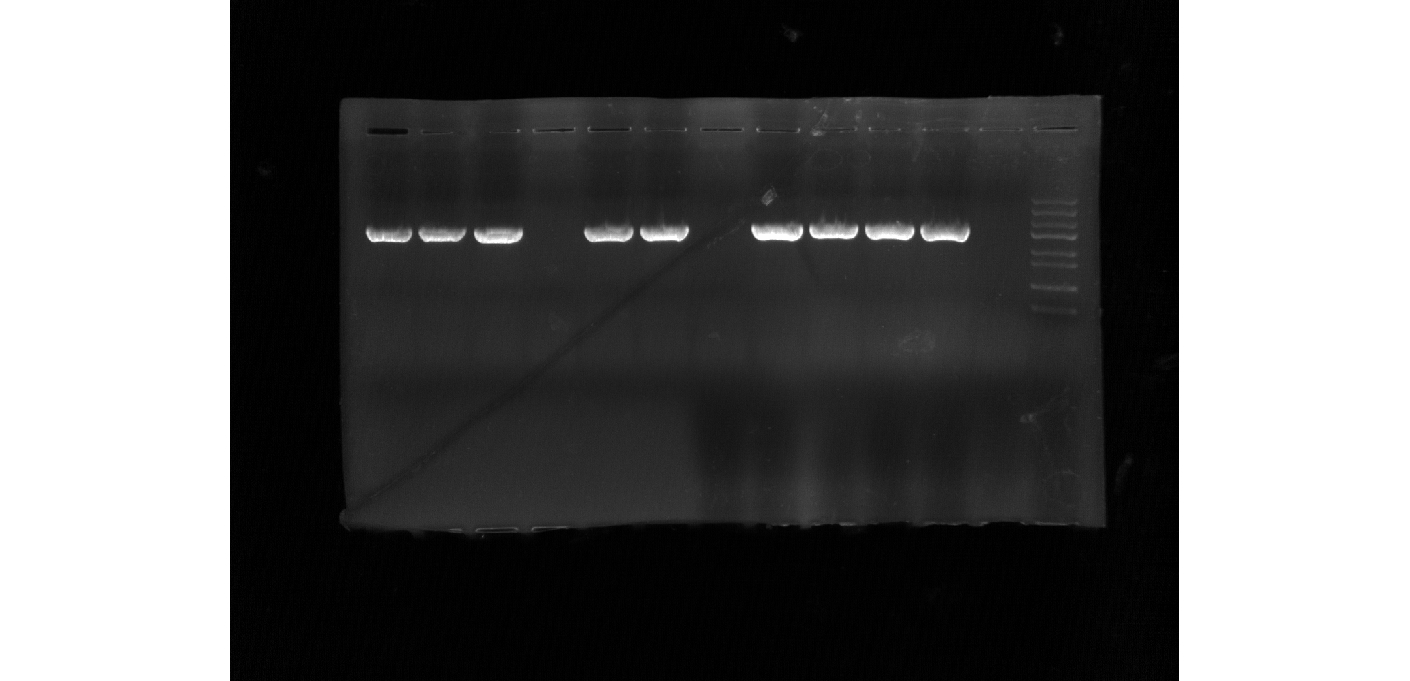


Supplementary Figure S7: Full uncropped original agarose gel images corresponding to Supplementary Figure S1(f).The gel was horizontally flipped for presentation in the main figure; this image shows the original orientation. Lane 13 contains the DNA molecular weight marker. All other lanes (except lane 5 and lane 7, which are blank controls) represent PCR products from replicate samples of the L. monocytogenes strain LM928Δ*hly*::*hly*. Additional lanes not shown in the main figure are also replicate samples of LM928Δ*hly*::*hly*, confirming consistent amplification of the target fragment across independent experiments.

(A)


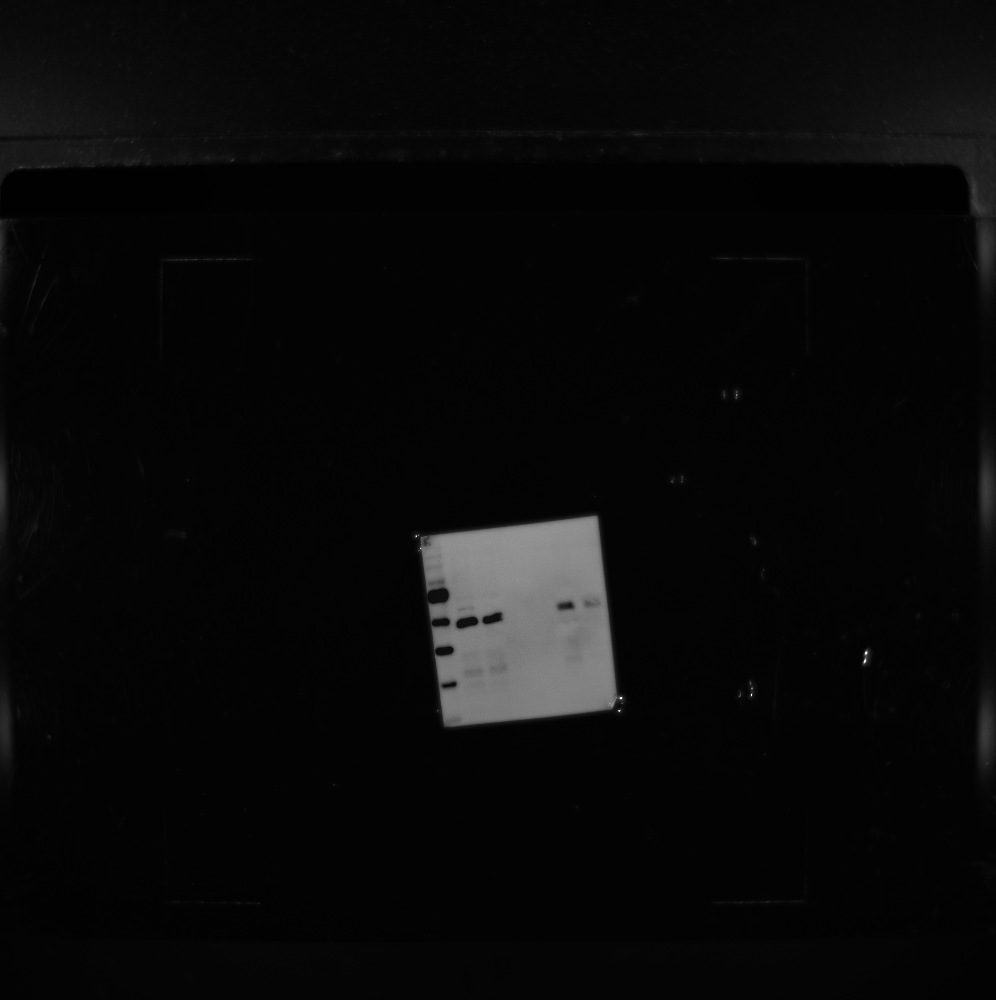


（A）


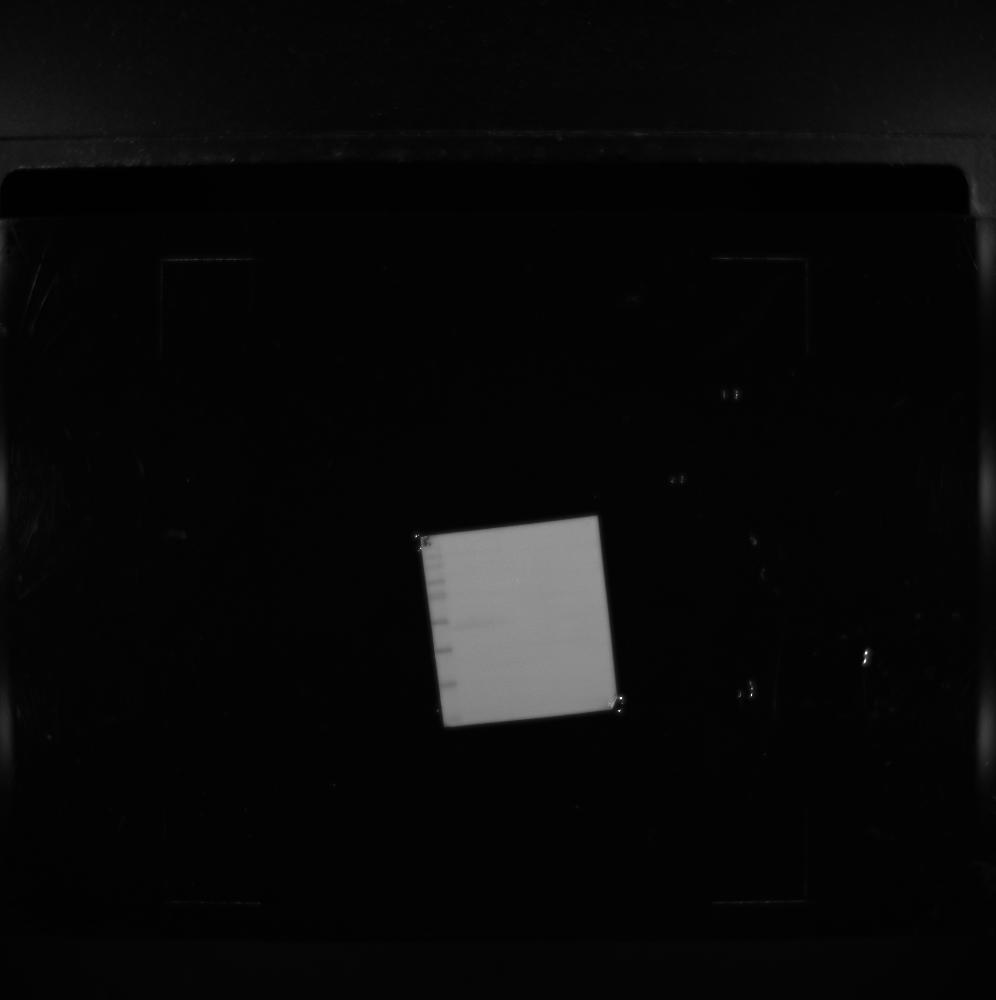


（B）


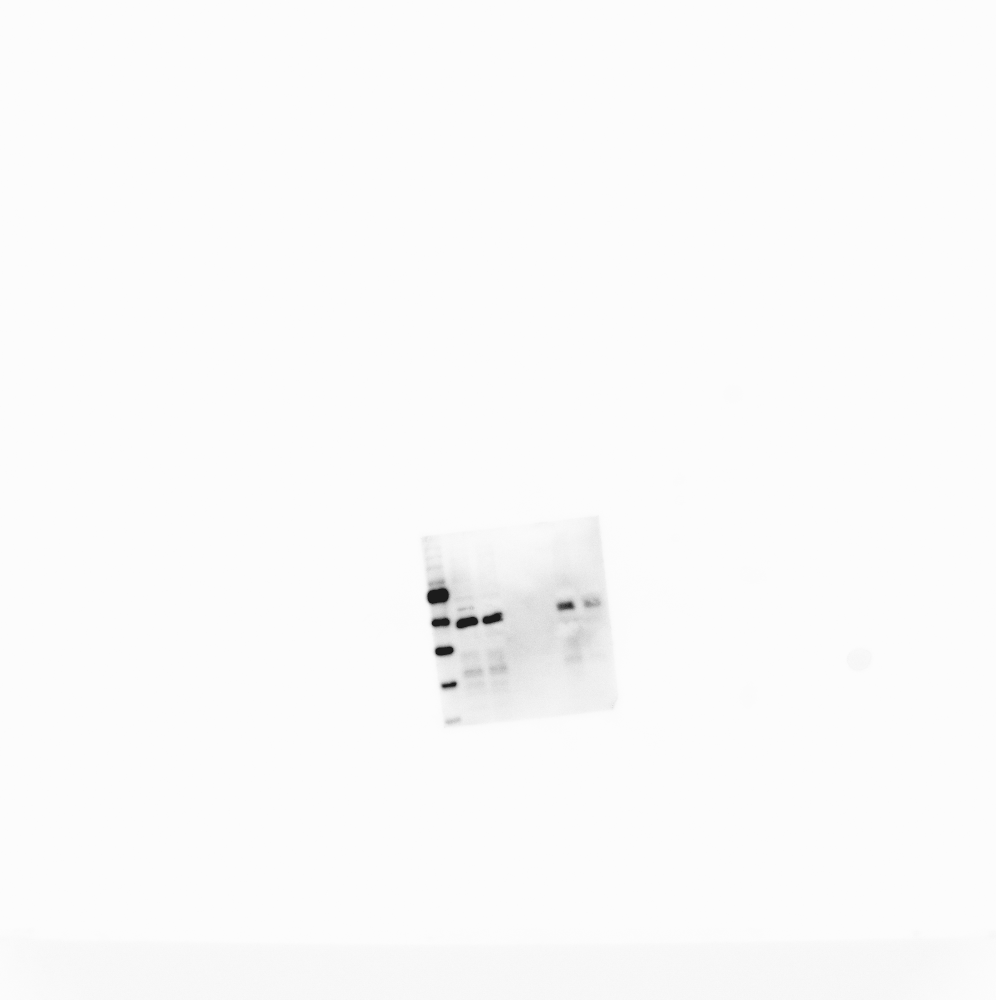


（C）

Supplementary Figure 8 Western blot analysis of LLO expression in total bacterial protein. (A) Brightfield image of the original uncropped membrane. (B) Standard chemiluminescence darkfield image of the uncropped membrane. (C) Enhanced exposure image of the uncropped membrane. Original protein marker is visible in the uncropped raw membrane. All sample loading quantities were calibrated based on BCA protein concentration measurement. This qualitative Western blot assay was conducted without an internal reference, because uniform protein loading was guaranteed by BCA quantification.


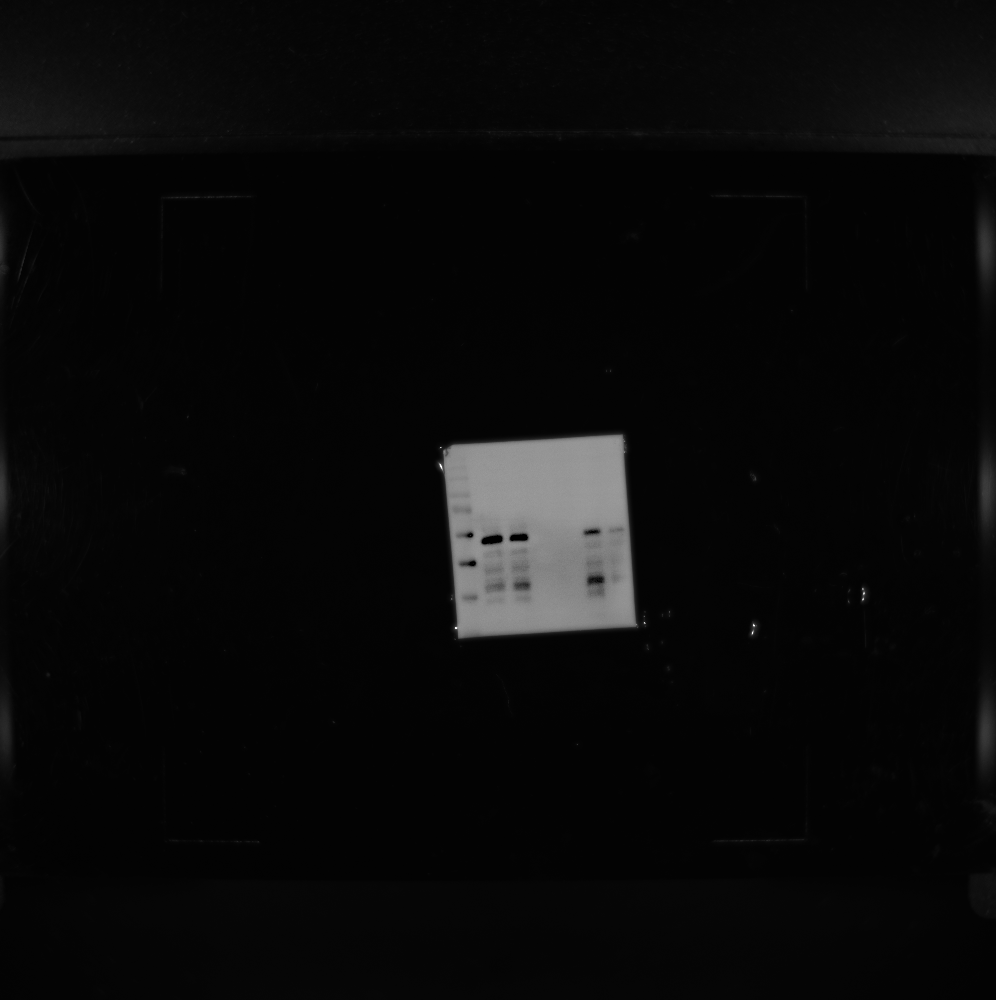


(A)


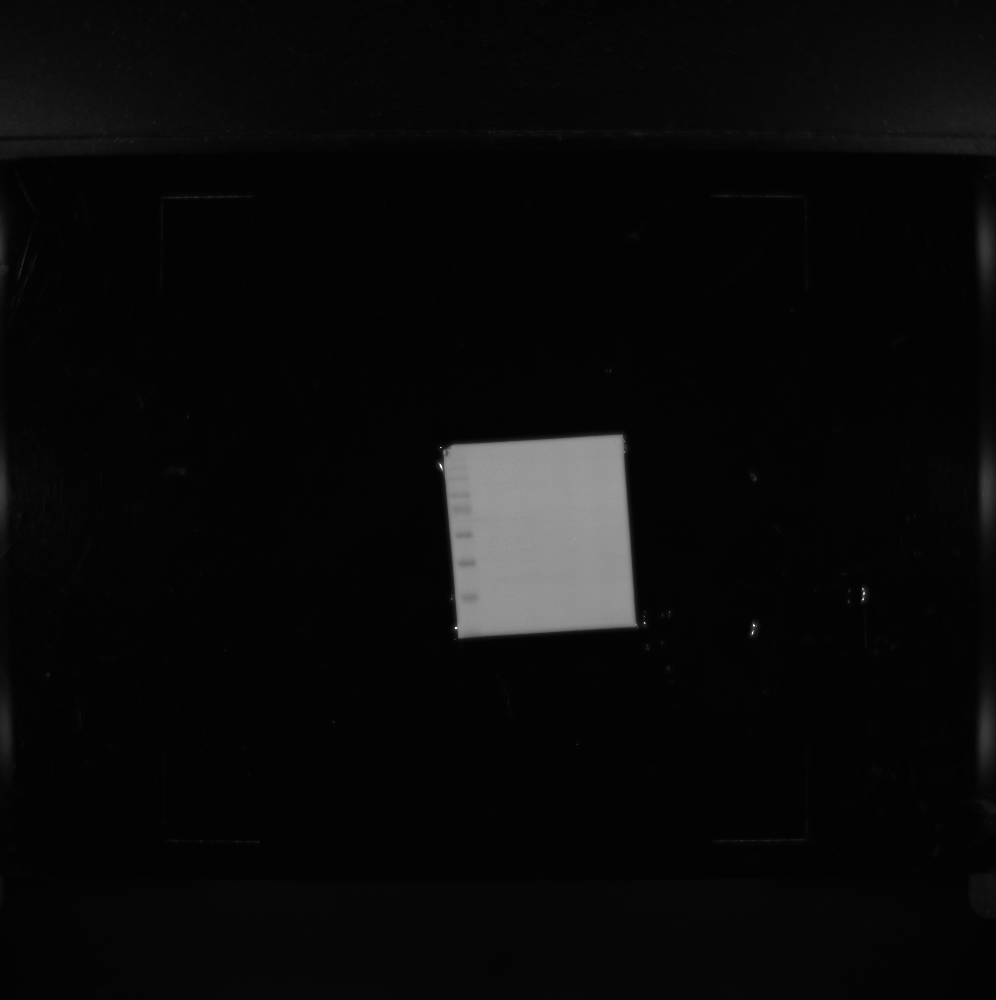


(B)


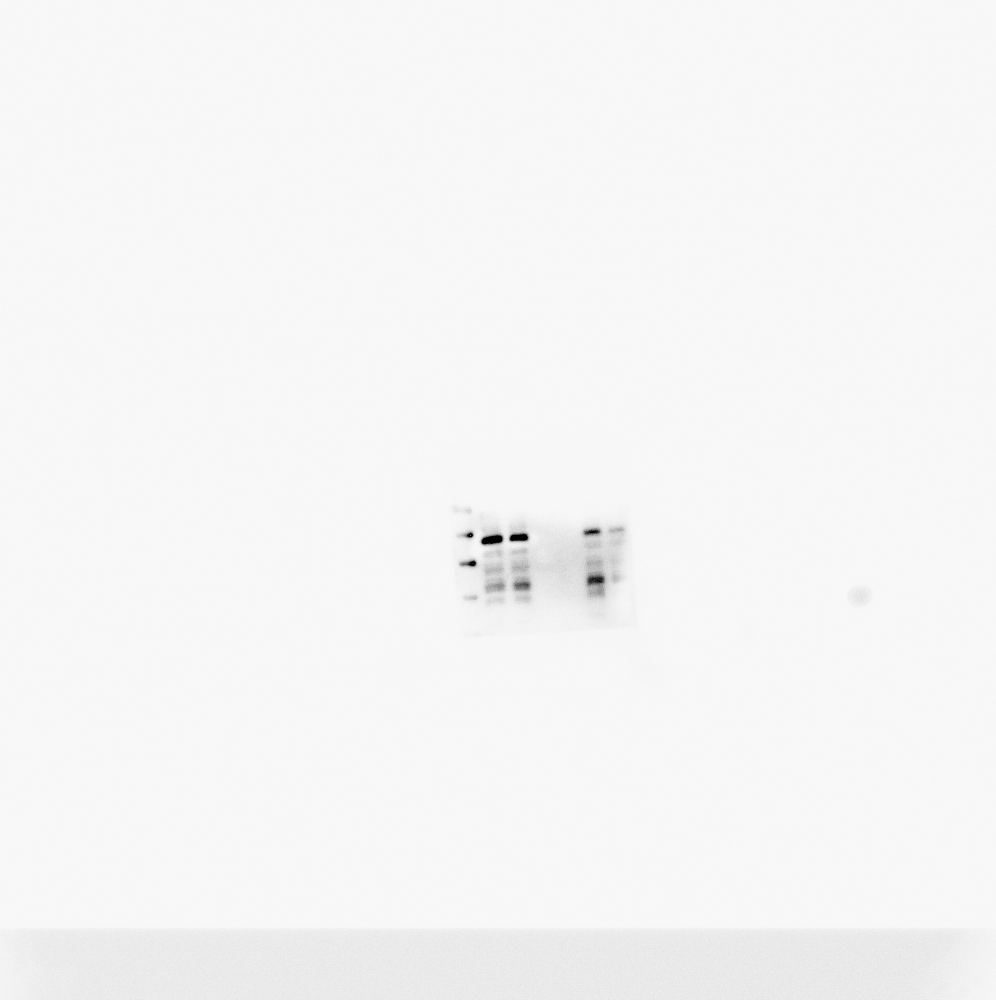


(C)

Supplementary Figure 9 presents Western blot detection of LLO in secretory protein.(A–C) are the brightfield view, standard chemiluminescence darkfield view and enhanced exposure view of the original uncropped membrane with intact protein marker. All secretory protein samples were quantified by BCA assay to ensure equal protein loading. No internal reference was applied in this qualitative assay, because consistent sample loading was strictly guaranteed through BCA concentration measurement.
